# Country-wide, multi-location trials of Green Super Rice lines for yield performance and stability analysis using genetic and stability parameters

**DOI:** 10.1038/s41598-024-55510-x

**Published:** 2024-04-24

**Authors:** Muhammad Shahzad Ahmed, Abid Majeed, Kotb A. Attia, Rana Arsalan Javaid, Faiza Siddique, Muhammad Shahbaz Farooq, Muhammad Uzair, Seung Hwan Yang, Asmaa M. Abushady

**Affiliations:** 1 Rice Research Program, Crop Sciences Institute, National Agricultural Research Center, Islamabad, Pakistan; 2 Department of Biochemistry, College of Science King Saud University, P.O. Box 11451, Riyadh, Saudi Arabia; 3 Food Science and Biological Engineering, Jiangsu University, Zhenjiang, 212013 Jiangsu People’s Republic of China; 4 National Institute for Genomics and Advanced Biotechnology (NIGAB), National Agriculture Research Centre (NARC), Park Road, Islamabad, Pakistan; 5 Department of Biotechnology, Chonnam National University, Yeosu, 59626 Republic of Korea; 6 Biotechnology School, 26th of July Corridor, Nile University, Sheikh Zayed City, 12588 Giza Egypt; 7 Department of Genetics, Agriculture College, Ain Shams University, Cairo, Egypt

**Keywords:** Rice, Stability, G × E interaction, GGE biplot, Paddy yield, Ecology, Plant sciences, Ecology

## Abstract

Rice (*Oryza sativa* L.) is an important member of the family *Poaceae* and more than half of world population depend for their dietary nutrition on rice. Rice cultivars with higher yield, resilience to stress and wider adaptability are essential to ensure production stability and food security. The fundamental objective of this study was to identify higher-yielding rice genotypes with stable performance and wider adaptability in a rice growing areas of Pakistan. A triplicate *RCBD* design experiment with 20 Green Super Rice (GSR) advanced lines was conducted at 12 rice growing ecologies in four Provinces of Pakistan. Grain yield stability performance was assessed by using different univariate and multivariate statistics. Analysis of variance revealed significant differences among genotypes, locations, and G x E interaction for mean squares (*p* < 0.05) of major yield contributing traits. All the studied traits except for number of tillers per plant revealed higher genotypic variance than environmental variance. Broad sense heritability was estimated in the range of 44.36% to 98.60%. Based on ASV, ASI, *bi,* Wi^2^, σ^2^_i_ and WAAS statistics, the genotypes G1, G4, G5, G8, G11 and G12 revealed lowest values for parametric statistics and considered more stable genotypes based on  paddy yield. The additive main effects and multiplicative interaction (AMMI) model revealed significant variation (*p* < 0.05) for genotypes, non-signification for environment and highly significant for G × E interaction. The variation proportion of PC1 and PC2 from interaction revealed 67.2% variability for paddy yield. Based on ‘mean verses stability analysis of GGE biplot’, ‘Which-won-where’ GGE Biplot, ‘discriminativeness *vs.* representativeness’ pattern of stability, ‘IPCA and WAASB/GY’ ratio-based stability Heat-map, and ranking of genotypes, the genotypes G1, G2, G3, G5, G8, G10, G11 and G13 were observed ideal genotypes with yield potential more than 8 tons ha^−1^. Discriminativeness *vs.* representativeness’ pattern of stability identifies two environments, E5 (D.I Khan, KPK) and E6 (Usta Muhammad, Baluchistan) were best suited for evaluating genotypic yield performance. Based on these findings we have concluded that the genotypes G1, G2, G3, G5, G8, G10, G11 and G13 could be included in the commercial varietal development process and future breeding program.

## Introduction

Rice (*Oryza sativa* L.) is an important member of the family Poaceae, along with wheat (*Triticum aestivum* L.) and maize (*Zea mays* L.) and; it is among one of the three crops on which human beings depend for their dietary nutrition. Rice is currently the most significant crop and a staple food in east Asia and many other regions worldwide^1,2^. Rice is cultivated in over 100 countries and the area under rice cultivation is about 164 million hectares with a production of 510 million metric tons^3^. Rice consumption has been slightly increased in the world during the last couple of years as global intake of rice was around 502 million metric tons during 2020–21, whereas in 2008–2009 it was around 437 million metric tons^4^. Per capita consumption of rice is 45 kg per annum^5,6^.

Rice is the second most important staple food, and Pakistan earn almost 2.5 billion US$ every year^7,8^. The rice share in agriculture value addition is 1.9 percent and its contribution in the total GDP of Pakistan is 0.4 percent. During the year 2020–2021, the area under rice cultivation was 3.5 million hectares, and the total rice production was 9.3 million tons for both coarse and Basmati rice^9^. Whereas, during the year 2023 there was a decline in area of production by 15 percent and yield loses by 21 percent due to high input prices and flood damages^10^. Production of rice is expected to increase every year to ensure food security as the world’s population is increasing and the rice demand is also increasing^11^. During 2050, world population is expected to be more than 10 billion and more food would be required to meet the food security challenges Click or tap here to enter text^12^. Rice crop faces number of challenges that hinder its production i.e., flood, drought, diseases and insect’s infestation, heat stress during pollination, cost of production, and availability of quality seed^13^.

Pakistan imports almost five thousand tons of hybrid rice seed every year from China and other sources; and almost 30 thousand tons of hybrid seed is produced locally by private companies. Seed production of locally adopted open-pollinated varieties is very less in the country to meet the farmers’ demand^14^. Due to unavailability of improved varieties and quality seed, poor farmers use leftover seed repeatedly that is a major cause of low yield. Under these circumstances, the development of improved rice breeding materials and their subsequent testing over numbers of locations across the region / country becomes prerequisite to recognize rice genotypes having higher yield potential, stable performance, and stress resilience value.

Rice production enhancement on a sustainable basis by using Green Super Rice (GSR) concept was proposed for rice breeding and production for rapid accelerating the development in rice functional genomics. As a result of collective efforts of Scientists, many rice cultivars have been developed with enhanced multiple biotic & abiotic stress tolerance, water & nutrient used efficiency and now these cultivars have been released in many countries in Asia and Africa^15^. Green Super Rice has characteristics of higher nutrient uptake efficiency, judicious use of water, resistance to economically important diseases (BLB, BLS etc.), insect pests and abiotic stresses specially drought, higher yield potential and good end use quality^16^. Stable performance of cultivars in terms of paddy yield and adaptability over a broad range of ecologies is a pre-requisite. Green Super Rice germplasm was acquired from China and IRRI in Pakistan through Pakistan Agricultural Research Council’s coordination system and evaluated at country wide Multilocation trials during the year 2021 by Rice Program, Crop sciences Institute, National Agricultural Research Center, Islamabad, Pakistan for assessing stability and adaptability of best- performing genotypes over a broad range of agro-ecologies.

Stability and adaptability analysis are important concepts in the study of rice genetics and breeding. Stability analysis is used to identify rice varieties that perform consistently better across different environments or growing conditions. This is important because rice is grown in diverse agro-ecological zones and climatic conditions. Stability analysis is conducted using statistical methods like regression analysis, analysis of variance (ANOVA) and AMMI (additive main effects and multiplicative interaction) model. AMMI model has shown to be quite helpful in analyzing multi-location yield trials in rice^17,18^. Multilocation yield trials (MLYTs) are widely used to assess the yield performance of rice germplasm in multiple environments. In MLYTs, the genotype into environmental interaction (GEI) is a main source of variation, which can be due to both genetic and non-genetic factors. The model is a type of analysis of variance (ANOVA) that separates the sources of variation in the data into additive and multiplicative components. The additive component of variance is due to the presence of main effects of the genotypes and environments and multiplicative component represents the interaction of genotypes and environments. The genetic variance can be estimated from the additive component of the model. Another commonly used method is the genotype plus genotype-by-environment interaction (GGE) biplot analysis. It is a graphical tool that visualizes the patterns of GEI and identifies the genotypes that perform well across environments^19,20^.

There are various statistical tools and packages that are used to perform stability analysis and G x E interaction estimation. The METAN R package is a commonly used tool for analyzing data from multilocation yield trials (MLYTs)^21^. The package provides functions for analyzing genotype by environment interaction (GEI) and for estimating the stability and adaptability of genotypes across environments. The METAN R package uses a mixed-effects model to estimate the genetic and environmental effects on the performance of genotypes in MLYTs. The model accounts for both the mean and the variance of the genotypes across environments. The package also provides functions for visualizing the patterns of GEI and to identifying stable yield performing genotypes^21^. Several studies have been conducted in MLYTs in rice and data was subjected to METAN R package for stability analysis and GEI analysis^22,23^. Present study aimed to scale out suitable genotypes that have stable yield performance in a broad range of rice-growing ecologies in Pakistan; that have the potential to become a variety for general cultivation by farmers.

## Material and methods

### Rationale of the study

Chinese scientists launched the Green Super Rice (GSR) project in 2005^24^ in response to the rising concerns about scarce resources, environmental pollution, and ecological destruction. The primary objective of research was to grow new rice strains with ecologically favorable traits such as insect and disease resistance, responsive to fertilizers effectively and drought tolerant^25^. Now, the GSR idea includes more than just creating new types with eco-friendly characteristics, which exemplifies the larger philosophy of crop breeding technology that emphasizes resource conservation and environmental friendliness. It also entails implementing risk-free, secure, effective, and high-yield crop management techniques^15,16^. The GSR project has been designed with five aims for GSR breeding^1,2^: creation of whole-genome selection platforms, combining environmentally favorable genes, involves developing novel germplasms with greater tolerance to biotic pressures as well as improved resistance to a variety of abiotic challenges, producing new sustainable GSR cultivars which includes hybrid and inbred types, increase grain yields and grain quality, these features are combined and lastly, the creation of field management strategies for higher production tailored for GSR. Therefore, considering these major focuses of GSR project, we designed this study with following objectives to have the tentatively available GSR genotypes for specific adaptability mechanisms in different ecological areas of Pakistan: 1) Evaluation and screening of drought-resistant and water-saving genotypes with better yield and quality under water deficit conditions; 2) Assessment of genotypes with better nutrient-use efficiencies with better growth, yield, and quality under reduced fertilizer application; 3) Evaluation of genotypes with better resistance to insects and pests under conditions of reduced application of pesticides; and 4) screening to the genotypes with more tolerance to abiotic stress such as salinity, heat, cold etc. with stable grain yield and quality.

### Plant materials and study sites description

**Figure 1:**
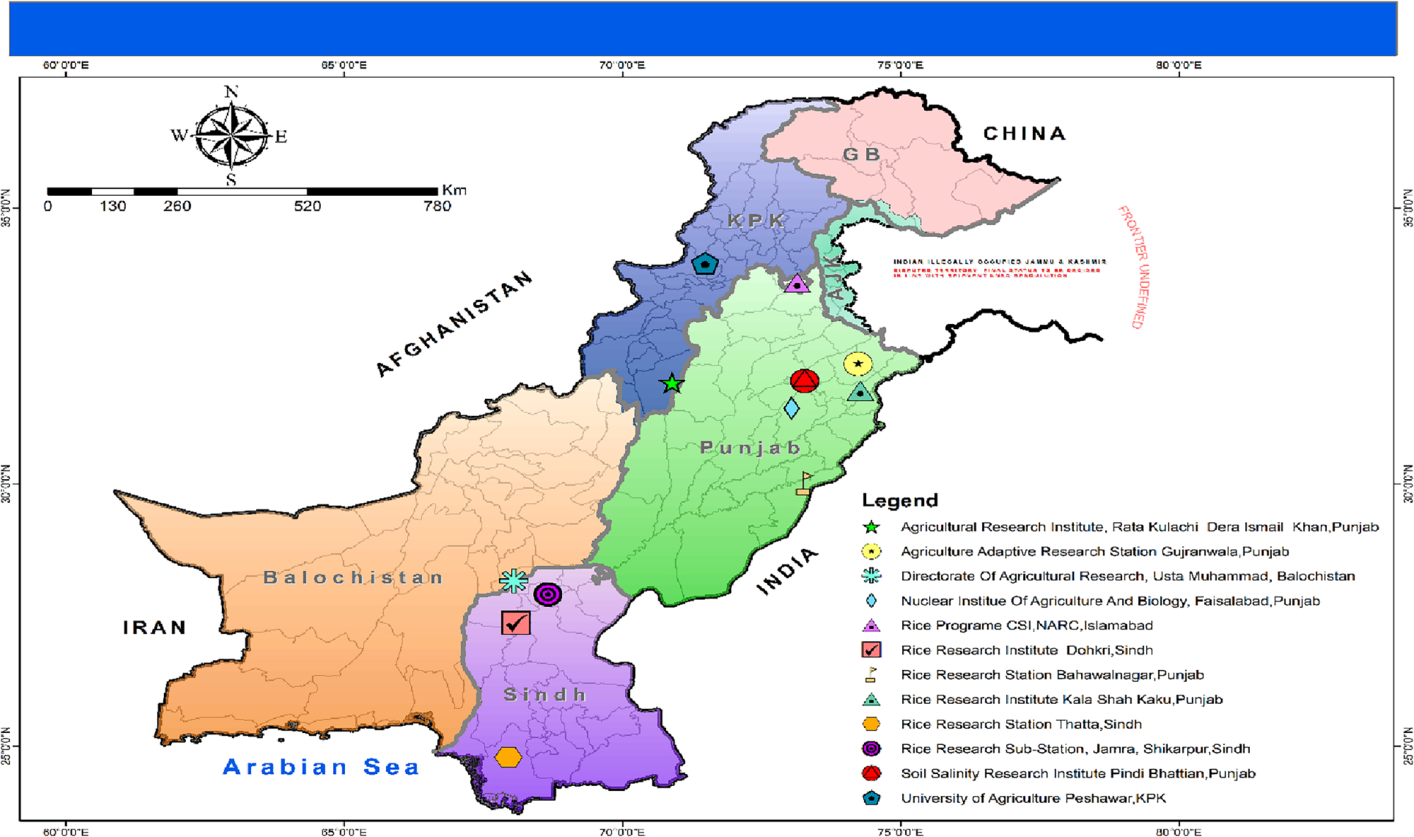
Map of experimental sites across Pakistan with legends indicating the locations of the Institutes where experiment was conducted. (ArcGIS-10.8 Software was used to generate the map^1^).

In the current study, screening of the adaptability mechanisms of eighteen GSR experimental genotypes with different growth durations comparing with two checks were conducted at twelve ecological locations across three provinces of Pakistan (Table 1; Fig. 1). The experiment was conducted using a randomized complete block design (RCBD) with three replications in 2021. Table 1 presents the ecological location, geography and environmental conditions which prevailed during the experimental season whereas Table 2 is represents the details of genotypes selected in this experiment with accession numbers, parentage, and source of germplasm. The rice genotypes and check cultivars selected for this experiment are mentioned in Table 2.

**Table 1:** Geographical details of locations for trials evaluation during the year 2021.

Environments codes	Locations	Temperature and rainfall pattern during rice growing season 2021												Soil type	Latitude and longitude
		June		July		August		September		October		November			
		Temp (◦C)	Rain (mm)	Temp (◦C)	Rain (mm)	Temp (◦C)	Rain (mm)	Temp (◦C)	Rain (mm)	Temp (◦C)	Rain (mm)	Temp (◦C)	Rain (mm)		
E1	Rice Program CSI, National Agricultural Research Center, Islamabad	29.9	4.09	30	9.61	29.1	4	28.3	7.42	22.5	6.35	16.0	0.000333	Loam	33.676380° N, 73.133190° E
E2	PARC-Rice Station Kala shah Kaku, Punjab	33.2	0.58	32.1	3.88	31.5	4.67	29.2	5.89	26.5	1.94	20.0	0.000333	Silty clay	31.726450° N, 74.266154° E
E3	Nuclear Institute of Agriculture and Biology, Faisalabad, Punjab	32.9	0.63	32.6	5.74	32.7	0.96	30.8	0.31	26.6	0.45	19.6	0	Loamy silt	31.398900° N, 73.033100° E
E4	Soil Salinity Research Institute Pindi Bhattian, Punjab	41.0	56.4	35	69	36	55	34	27	30	5	24	2	Sandy loam/salty	31.8950° N 73.27060° E
E5	Agricultural Research Institute, Rata Kulachi Dara Ismael Khan, Khyber Pakhtunkhwa	33.9	1.5	33.8	1.9	33.1	1.4	32	0.5	27.4	0.0	20.1	0	Sandy/loamy sand	31.872015° N, 70.884298° E
E6	Directorate of Agricultural Research, Usta Muhammad, Baluchistan	37.7	0.54	38.0	1.22	36.1	5.08	32.27	0.11	26.53	16.11	19.0	2.8	Alluvial soil, silt loam	28.189970° N, 68.043894° E
E7	Rice Research Station, Bahawalnagar, Punjab	33.6	2.03	34.5	0.39	34.3	0	31.3	0.57	28.2	0.55	20.7	0	Loamy or sandy loam	30.0128° N, 73.2715° E
E8	Rice Research Institute Dohkri, Sindh	39.1	6.2	38.0	56.6	35.0	82.3	33.5	29.8	30.4	3.2	25.3	2.3	Sandy clay loam	27.376751° N, 68.081996° E
E9	Rice Research Station, Thatta, Sindh	38.8	8.44	36.6	44.4	34.8	79.4	34.8	38.1	34.6	2.7	28.6	3.3	Silty clay	24.77780° N, 67.94790° E
E10	Agriculture Adaptive Research Station, Sheikhupura, Punjab	44.0	19.5	42.2	51.0	39.6	44.6	38.1	33.4	34.3	2.5	26.3	2.8	Clay loam	31.71666° N, 73.98502° E
E11	Agriculture Adaptive Research Station, Gujranwala, Punjab	31.8	1.07	30.8	9.85	31	1.95	29.1	2.67	25.2	1.7	18.4	0.03	Silty clay loam	32.2042° N, 74.2306° E
E12	Rice Research Sub-Station Jamara, Shikarpur, Sindh	38.5	8.3	36.3	44.0	34.5	78.8	34.5	37.8	34.3	2.7	28.4	3.3	Silt loam	27.9216° N, 68.6547° E

**Table 2:** Genotypes names, their codes, gene bank-accession numbers, source of germplasm and source of germplasm.

S. No	Genotypes	Codes	Accession numbers	Source of germplasm	Original source
1	NRPC-1	G1	32156	PGRI, BCI NARC, PAK	IRRI
2	NRPC-2	G2	32161	PGRI, BCI NARC, PAK	IRRI
3	NRPC-3	G3	32163	PGRI, BCI NARC, PAK	IRRI
4	NRPC-4	G4	32164	PGRI, BCI NARC, PAK	IRRI
5	NRPC-5	G5	32165	PGRI, BCI NARC, PAK	IRRI
6	NRPC-6	G6	32168	PGRI, BCI NARC, PAK	IRRI
7	NRPC-7	G7	32169	PGRI, BCI NARC, PAK	IRRI
8	NRPC-8	G8	32170	PGRI, BCI NARC, PAK	IRRI
9	NRPC-9	G9	32171	PGRI, BCI NARC, PAK	IRRI
10	NRPC-10	G10	32172	PGRI, BCI NARC, PAK	IRRI
11	NRPC-11	G11	32176	PGRI, BCI NARC, PAK	IRRI
12	NRPC-12	G12	32178	PGRI, BCI NARC, PAK	IRRI
13	NRPC-13	G13	32179	PGRI, BCI NARC, PAK	IRRI
14	NRPC-14	G14	32183	PGRI, BCI NARC, PAK	IRRI
15	NRPC-15	G15	32219	PGRI, BCI NARC, PAK	IRRI
16	NRPC-16	G16	32220	PGRI, BCI NARC, PAK	IRRI
17	NRPC-17	G17	37586	PGRI, BCI NARC, PAK	IRRI
18	NRPC-18	G18	37588	PGRI, BCI NARC, PAK	IRRI
19	KSK-434	G19		RRI, KSK, Lahore	Pakistan
20	IR-06	G20		RRI, KSK, Lahore	Pakistan

PGRI, BCI NARC; Plan Germplasm Research Program, Bioresources Conservation Institute, National Agricultural research Center,

### Agronomic and cultural practices

Considering the climatic conditions of each study site, during the first week of June, 2021, a 100-g pre-treated seed of GSR genotypes with two genotypes of checks was sown for nursery through dry method of nursery sowing on 2 ft^2^ plot size^24^. The plots were labelled with genotype names and codes. Depending on the prevailing climatic conditions, a 30 days-old nursery was shifted to paddy transplantation fields at the respective study location, where the transplantation was done manually. Transplantation of all selected genotypes was done between the 1st to 10th of July through a common straight-row technique comprising three replications at each study site. The plot size of the transplanted seedlings was maintained at 2.0 m × 1.0 m with five rows containing eight seedlings per row. The line-to-line and plant-to-plant spacing was maintained at 20 cm within the plot. To measure the variation in the genetic components, the grain yield and yield-related attributes were estimated at the physiological maturity stage during which five randomly selected seedlings were taken in each replicated plot. Plant height and panicle length measurements were carried out with standard designated methods^26^. The data for the number of productive tillers, grains per panicle, 1000-grain weight and grain yield was calculated with standard agronomic procedures^27^. To fulfil the crop nitrogen (N) requirements, synthetic Urea fertilizer was applied in three equal splits viz-10, 40, and 65 days of seedling transplantation. Whereas phosphorus (P) and potash (K) required dosages were applied in the form of diammonium phosphate (DAP), and muriate of potash (MOP) after 10 days of transplantation. All the N, P and K fertilizer management was done at the recommended dosage of 150, 90, and 60 kg/ha, respectively, which marginally varied based on the locality. Weeds were removed manually two times through hand-weeding as well as through recommended pesticides^28^.

### Statistical tools

#### Analysis of variance

The morphological data for all the experimental genotypes at selected localities was subjected to the combined analysis of variance (ANOVA) through R software (4.1.3 version) (https://cran.r-project.org/bin/windows/base/old/4.1.3/). Additionally, ANOVA-based data was subjected for further evaluation of the effects of genotypes (G), environments (E), and replications (R) and determining the interaction magnitude of the G × E. ArcGIS-10.8 Software was used to generate the map^29^.

#### Genetic components

The mean sum of squares of all the genotypes was used to determine the genetic and environmental effects for different traits^30^. For each specific trait, the average of the sample data was taken to have replication mean values^31–33^. The average data among all traits was analyzed statistically^34,35^ as well as biometrically^36,37^.

- **Genotypic and phenotypic variance**

$${\sigma }^{2}g=\frac{{\text{GMS}}-{\text{EMS}}}{{\text{r}}}$$

where GMS refers to the genotype mean square, EMS designates the error mean square, and r denotes the replications.

$${\sigma }^{2}p={\sigma }^{2}g+{\sigma }^{2}e$$

Here ${\sigma }^{2}p$ represents phenotypic variance, ${\sigma }^{2}g$ is denots the genotypic variance, and ${\sigma }^{2}e$ designats towards the environmental variance.

- **Environmental variance**

$${\sigma }^{2}e=\frac{{\text{EMS}}}{{\text{r}}}$$

Here, ${\sigma }^{2}e$ denotes environmental variance, EMS designates towards error mean square, and r represents the replication number.

- **H**^**2**^

$${h}_{B}^{2}=\frac{{\sigma }^{2}g}{{\sigma }^{2}p}$$

where ${h}_{B}^{2}$ denots the broad-sense heritability, whereas ${\sigma }^{2}g$ represents genotypic variance and ${\sigma }^{2}p$ is the phenotypic variance.

#### Calculation of stability statistics

Stability prediction among GSR genotypes and assessment of the yield components across different locations, the univariate and multivariate stability analyses were conducted because of the possible occurrence of substantial variations among different environments.

#### Univariate stability analysis

Univariate stability analysis was performed for the above-mentioned yield and yield-related components of all genotypes by using AMMI Stability Value (ASV)^38,39^ and AMMI Stability Index (ASI)^40^, Shukla’s stability variance (σ^2^)^41^ and Wricke’s ecovalence (Wi^2^)^42^.

AMMI stability value (ASV)

$$ASV=\sqrt{ {\left(\frac{{SS}_{IPCA1}}{{SS}_{IPCA2}} \left(IPCA1\right)\right)}^{2}+{\left(\left(IPCA2\right)\right)}^{2}}$$

${{\text{SS}}}_{{\text{IPCA}}1}$ and ${{\text{SS}}}_{{\text{IPCA}}1}$ denote the sum of squares while IPCA1 and IPCA2 represent the scores of genotypes in the first and second principal component interactions, respectively^39^.

- **AMMI stability index (ASI)**

AMMI-model based AMMI Stability Index (ASI) was designed by^40^ to calculate the stability among genotypes by the following equation:

$$ASI=\sqrt{ \left[{(IPCA1 \times {\theta }_{1}^{2})}^{2}+{(IPCA2 \times {\theta }_{2}^{2})}^{2}\right]}$$

IPCA1 and IPCA2 represent the values of the first and second principal component interactions, whereas, and ${\uptheta }_{1}^{2}$ and ${\uptheta }_{2}^{2}$ show the percentage sum of square descripted by these interactions.

- **Regression coefficient (** ***b***_***i***_ **)**

Regression coefficient was estimated according to Eberhart and Russell^43^. Here ***b***_***i***_ is equal to 1. The genotypes not equal to 1 are sensitive to environmental change.

- **Wricke’s ecovalence**

The overall participation of respective genotype to the sum of squares of G × E is evaluated by the ecovalence parameter analysis^42^ as given following:

$${W}_{i}^{2}={\sum ({X}_{ij}-\overline{{X }_{i.}}-\overline{{X }_{.j}}-\overline{{X }_{..}})}^{2}$$

In this context, ${X}_{ij}$ represents the average of genotype “i” in environment “j”, additionally, $\overline{{X }_{i.}}$ refers to the mean grain yield of genotype “i”, ${X}_{.j}$ signifies the average yield of the *j*th environment, and ${X}_{..}$ corresponds to the overall grand mean.

- **Shukla’s stability variance**

The concept of Shukla's stability variance was introduced by^41^. It aims to evaluate the variability of genotypes across diverse climatic conditions and is defined using the following equation:

$${\sigma }^{2}=\left[\frac{p}{(p-2)(q-1)}\right]{W}_{i}^{2}-\frac{\sum {W}_{i}^{2}}{(p-1)(p-2)(q-1)}$$

where p and q represent the numbers of genotypes and environments, respectively, and ${W}_{i}^{2}$ is the Wricke’s ecovalence of the *i*th genotype.

- **Weighted average of absolute score (WAASBY)**

To quantify the genotypic stability, we used the function WAASB from METAN R package^21^ to compute the WAASB index^44^, as follows:

$${WAASB}_{i}=\frac{\sum_{k=1}^{p}\left|{IPCA}_{ik}\times {EP}_{k}\right|}{\sum_{k=1}^{p}{EP}_{k}}$$

where ${WAASB}_{i}$ represents the weighted average of absolute scores for the *i*th genotype; while ${IPCA}_{ik}$ denotes the score of the *i*th genotype in the *k*th interaction principal component axis (IPCA); and ${EP}_{k}$ signifies the amount of variance explained by the *k*th IPCA. Genotypes with a lower WAASB value are considered to exhibit greater stability, while environments with higher WAASB values indicate higher genotypic variance.

#### Multivariate stability analysis

Multivariate stability analysis through AMMI^45^ and GGE biplot^46^ were conducted to evaluate the best genotypes respective to testing environments comprising the high stability and performance, thereby better understanding of the G × E.

#### AMMI model

In the present study, multivariate stability was evaluated using the Additive Main Effect and Multiplicative Interaction (AMMI) model to assess genotype *vs.* environment interaction and predict the stability of GSR genotypes. The AMMI model combines pooled ANOVA to assess additive main effects, followed by employing singular value decomposition (SVD) on the total error matrix to compute the interaction principal components (IPCs). We determined the AMMI model in R by utilizing the metan library (Olivoto & Lúcio, 2020). AMMI model. To compute the AMMI model, the following equation was used^45^:

$${Y}_{ij}=\mu +{\alpha }_{i}+{\beta }_{j}+{\sum }_{k=1}^{n}{\uplambda }_{k\gamma ik}{\delta }_{jk}+{\varepsilon }_{ij}$$

Here, ${Y}_{ij}$ denotes the mean values of performance of the *i*th genotype in the respective *j*th environmental conditions, ${\alpha }_{i}$ represents the fixed effect of the respective genotype, ${\beta }_{j}$ shows the environmental effect, n is the total number of IPCA hold in AMMI model, *λ*_*k*_ is individual value for IPC axis k, *γ*_*ik*_ represents the *i*th GSR genotype value of eigenvector for IPC axis k, ${\delta }_{jk}$ shows the eigenvector value for *j*th environment for IPC aaxis k, and lastly ${\varepsilon }_{ij}$ denotes the mean residual.

#### GGE biplot analysis

A study analysis was conducted for the sustainable phenotypic reliability of the different climatic conditions of the biplot graphic after rating the adaptable GSR genotypes^47^. The biplot graphs designed in this study represent the variables which impact the genotype, where the ordinate is the IPCA1. The genotype with IPCA1 falling near to zero would be considered an ideal and stable genotype, where low stability represents low productivity of the respective genotype (Gauch & Zobel, 1996; Kempton, 1984.

To compute different parametric and non-parametric stability statistics, we also used the web-based STABILITYSOFT tool^48^. The AMMI stability was used in conjunction with the simultaneous selection indices (SSI) technique, which is based on average yield and stability^49^. The average of collected data was analyzed graphically (Yan & Tinker, 2006 through R 4.1.3 software (https://www.r-project.org/) and GGE Biplot GUI tools (https://CRAN.R-project.org/package=GGEBiplotGUI). Currently, there could be several techniques to deliver the key findings: firstly, polygon biplot graphics to analyze how each genotype responded to specific environments across various experimental sites, revealing the GGE pattern. Secondly, the study focused on identifying the most suitable genotype(s) based on both mean performance and stability. Moreover, graphical displays of concentric circles with vectors of entries were used to unveil associations between different environmental factors and genotypes, with a particular emphasis on identifying stable genotypes.

### Ethical approval

The plant collection and use was in accordance with all relevant guidelines.

## Results and discussion

### Results

#### Mean performance and combined analysis of variance

The mean performance of genotypes at 12 locations is mentioned in Table 3. Combined analysis of GSR genotypes (Table 4) revealed significant differences for mean squares (*p* < *0.05*) for traits under study like Plant height (PH), number of tillers per plant (NT), panicle length (PL), grains per panicle (GPP), thousand grain weight (TGW) and paddy yield in Kg per hectare (PY. Kg ha^−1^). The mean squares for genotypes (G), environments (E) and genotypes x environmental interaction (G X E) for almost all the traits depicted significant differences (p < 0.05). Combined analysis of variance showed highly significant (*p* > *0.01*) differences for G X E interaction of genotypes for all the parameters under study, which means genotypes responded differently in some environments and need to be further tested for stability and adaptability analysis.

**Table 3:** Mean performance of GSR lines over 12 locations countrywide, pooled average yield and percent increase or decrease than local check cultivars.

	E1	E2	E3	E4	E5	E6	E7	E8	E9	E10	E11	E12	Avr. Paddy yield (kg/ha)	% > < L.C-I	% > < L.C-II
G1	9887	9683	5896	8300	4833	10321	5752	6826	6807	6317	6634	3913	7097	6.22	10.58
G2	9687	9233	6127	7117	4167	11776	3870	6582	7017	6477	6559	3048	6804	1.85	6.03
G3	9831	8517	6150	7392	3667	12144	4722	6641	7017	6113	6838	4077	6925	3.65	7.91
G4	9580	8850	6108	6558	4167	8745	4952	6894	6990	6050	6836	3872	6633	− 0.72	3.35
G5	8672	7958	5761	6083	3333	8502	5504	6948	7240	5980	6713	4201	6407	− 4.09	− 0.16
G6	9533	9625	6087	7500	4500	7430	5390	6497	7130	6210	6540	3707	6679	− 0.04	4.06
G7	9118	8008	5584	5683	3500	11795	4837	6670	7180	6080	6842	3039	6528	− 2.30	1.71
G8	7774	8167	5891	6275	3667	10448	4098	6929	7373	5987	6963	3212	6398	− 4.23	− 0.30
G9	10226	10800	5982	8300	5333	10531	5393	7033	7157	6573	6674	3871	7322	9.60	14.09
G10	9257	8283	6115	5958	5333	11330	6451	6266	7067	5517	6378	3962	6826	2.17	6.36
G11	9215	8408	5828	5567	4833	9741	6717	6477	6823	6050	6403	4135	6683	0.03	4.13
G12	7836	9483	5986	6767	4167	9460	5969	6584	6997	6387	6763	4110	6709	0.41	4.53
G13	7805	8917	6191	6067	4333	12505	5662	6488	6947	6327	6374	4720	6861	2.69	6.90
G14	8103	8750	6314	6675	4667	11598	5053	6718	6623	5717	6882	3089	6682	0.01	4.11
G15	6605	5875	4811	5058	3167	8618	5862	7338	7090	6147	7561	2998	5927	− 11.28	− 7.64
G16	9015	10633	6101	7933	3000	9432	5970	7915	7030	6027	8284	3212	7046	5.46	9.78
G17	8267	6633	5459	7075	3167	7259	5108	7064	6547	6397	6436	2636	6003	− 10.14	− 6.46
G18	8944	6408	5145	6133	3500	7636	6038	6083	6800	6566	6074	3954	6106	− 8.60	− 4.85
G19 (L.C.I)	8205	7717	5847	7883	4833	7357	6559	6531	7307	6497	6706	4736	6681	4.10	4.10
G20 (L.C.II)	8574	5517	4628	5633	5667	10263	6318	6622	6217	6420	6710	4448	6418	0.00	0.00

**Table 4:** Combines ANOVA of significant yield attributing trains of GSR lines on the basis of mean squares.

Source of variation	Degree of freedom	Plant height	Number of tillers	Panicle length	Grains per panicle	1000 grain weight	Paddy yield
Genotypes	19	2152.1*	40.96 ns	36.58*	4727*	61.88*	4,575,140*
Replications	2	10,219*	1.09*	10.49*	856*	5.06*	53,350,000*
Environments	11	6768.5 ns	439.73*	120.76*	102,783*	881.35*	189,000,000*
Genotypes × Environments (G × E)	209	158.5*	31.34*	7.40*	1259*	9.292**	2,049,467*
Error (REP × ENV × GE)	456	30.1	22.79	3.71	400	2.658	1,315,995

**Significance @0.1%, *significance @0.5%, ns; non-significant.

#### Estimation of genotypic and phenotypic components of variances

Analysis of genotypic and phenotypic variances for metric traits of GSR lines is represented in Table 5. Phenotypic variance has been partitioned into genotypic variance and environmental variance. In our study, all the traits except NT revealed higher genotypic variance than environmental variance. Broad sense heritability was estimated in the range of 44.36% to 98.60%. Maximum heritability value was recorded for PH (98.60%) while NT showed minimum value for heritability (44.36%). All other traits under study revealed higher values for heritability (> 70%). Higher genotypic variance and higher values for broad sense heritability depicts that parameters under study have additive genes and more stable characters for selection in the genotypes for variety development.

**Table 5:** Estimation of genetic components of GSR rice for yield and yield-related attributes.

Genetic parameters	Plant height	Number of tillers	Panicle length	Grains per panicle	1000 grain weight	Paddy yield
Genotypic variance	707.33	6.06	10.96	1442.33	19.74	1,086,381.67
Environmental variance	10.03	7.60	1.24	133.33	0.89	438,665
Phenotypic variance	717.37	13.65	12.19	1575.67	20.63	1,525,046.67
Heritability (Broad sense)	98.60	44.36	89.86	91.54	95.70	71.24

#### Univariate stability statistics

**Estimation of univariate parametric stability statistics**

The results of parametric stability statistics of yield and yield-related traits of 20 GSR lines are presented in Table 6. The parametric stability statistics like AMMI stability value (ASV), AMMI stability index (ASI), stability value based on regression coefficient (*bi*), Shukla’s stability variance (σ^2^_i_), Wricke’s ecovalence (Wi^2^) and weighted average of absolute scores (WAAS) were studied to assess the stable genotypes based on paddy yield. These parametric stability statistics were used based on concepts that genotypes that have a stability value near to zero (0) are more stable. Parametric stability statistics values were assessed for 20 genotypes over 12 locations country wide in four provinces (Punjab, Sind, Khyber Pakhtunkhwa, Baluchistan) and Islamabad, Federal Capital, in Pakistan. Based on ASV, ASI, *bi,* Wi^2^, σ^2^_i_ and WAAS statistics, the genotypes G1, G4, G5, G8, G11 and G12 revealed lowest values for parametric statistics and considered more stable genotypes on the bases of paddy yield than rest of other genotypes. Minimum ASV value revealed by genotypes i.e., G12 (2.26) and maximum value by G19 (13.6). The minimum value for ASI was depicted by G12 (8.09) followed by G11 (11.7) and maximum value by G19 (48.8). The lowest value for ***b***_***i***_ was revealed by G19 (0.54) and highest value for ***b***_***i***_ was recorded in G2 (1.38). Genotypes, G1, G4, G5, G8, G11 and G12 revealed lowest values for Wi^2^ (< 4 × 10^6^), σ^2^_i_ (< 4 × 10^5^) and WAAS (4.3 to 10.8).

**Table 6:** Parametric stability statistics of yield and yield related traits of GSR lines evaluated at 12 locations across Pakistan.

Genotypes	Univariate stability statistics for Paddy yield						
	ASV^(1)^	ASI^(2)^	*b*_*i*_^(3)^	*W*_*i*_^*2*4^	*σ*^2^_*i*_	WAAS	Ranking
G1	5.2	18.6	1.11	3,703,398	336,127.5	10.8	2
G2	11.7	42	1.38	8,528,254	823,486.7	18.2	7
G3	9.93	35.6	1.31	6,997,377	668,852.7	12.9	4
G4	4.4	15.8	0.98	2,522,387	216,833.5	8.18	13
G5	5.55	19.9	0.88	2,844,030	249,322.6	8.08	16
G6	11	39.5	0.86	8,883,773	859,397.8	21.4	12
G7	9.72	34.8	1.32	6,471,572	615,741	16.9	14
G8	4.59	16.5	1.11	3,814,730	347,373.2	9.51	17
G9	8.34	29.9	1.18	6,931,041	662,152.1	16.9	1
G10	6.8	24.4	1.03	5,714,189	539,237.8	15.1	6
G11	3.26	11.7	0.90	3,696,320	335,412.6	8.08	9
G12	2.26	8.09	0.91	2,709,855	235,769.7	4.3	8
G13	11.3	40.7	1.10	9,553,098	927,006.3	18.7	5
G14	8.73	31.3	1.16	5,181,614	485,442.2	12.6	10
G15	11	39.4	0.77	12,951,290	1,270,258	24.7	20
G16	8.26	29.6	1.21	10,549,380	1,027,641	15.9	3
G17	10.7	38.3	0.82	8,317,319	802,180.2	16.9	19
G18	11.1	39.8	0.72	8,019,729	772,120.5	18.3	18
G19 (L. C-I)	13.6	48.8	0.54	11,113,063	1,084,579	19.2	11
G20 (L. C-II)	12	42.9	0.71	14,277,088	1,404,177	20.9	15

ASV: AMMI Stability Variance; ASI: AMMI Stability Index; FA: Stability Measure Based on Fitted AMMI Model; WAAS: Weighted average of absolute scores.

#### Multivariate stability statistics

##### AMMI analysis of variance

The additive main effects and multiplicative interaction (AMMI) model was used to assess the genotype × environmental interaction (G × E) of 20 GSR lines over 12 locations for paddy yield that revealed significant variation (p < 0.05) for genotypes and non-signification for environment and highly significant for G × E interaction (Table 7). The variation proportion of PC1 and PC2 from interaction revealed 67.2% variability for paddy yield.

**Table 7:** AMMI analysis of variance for grain yield of 20 GSR lines cultivated across the country at 12 locations.

Source	Df	Mean Sq	F value	Pr (> F)	Proportion	Accumulated
ENV	11	1.89e + 08	5.20	3.63e-04	NA	NA
REP(ENV)	24	3.63e + 07	27.61	1.32e-73	NA	NA
GEN	19	4.58e + 06**	3.48	1.37e-06	NA	NA
GEN:ENV	209	2.05e + 06*	1.56	5.85e-05	NA	NA
PC1	29	5.80e + 06**	4.40	0.00e + 00	39.2	39.2
PC2	27	4.43e + 06**	3.37	0.00e + 00	27.9	67.2
PC3	25	2.22e + 06	1.69	2.08e-02	13.0	80.1
PC4	23	1.38e + 06	1.05	4.00e-01	7.4	87.5
PC5	21	8.39e + 05	0.64	8.89e-01	4.1	91.7
PC6	19	7.33e + 05	0.56	9.33e-01	3.3	94.9
PC7	17	6.01e + 05	0.46	9.69e-01	2.4	97.3
PC8	15	3.18e + 05	0.24	9.99e-01	1.1	98.4
PC9	13	3.00e + 05	0.23	9.98e-01	0.9	99.3
PC10	11	1.83e + 05	0.14	1.00e + 00	0.5	99.8
PC11	9	9.88e + 04	0.08	1.00e + 00	0.2	100.0
Residuals	456	1.32e + 06	NA	NA	NA	NA
Total	928	4.84e + 06	NA	NA	NA	NA

*Variables with significant (*p* < 0.05) genotype-versus-environment interaction. **Significance @0.1%, *significance @0.5%, ns; non-significant.

##### Mean *verses* stability analysis of GGE (genotype + genotype × environment) biplot

**Figure 2:**
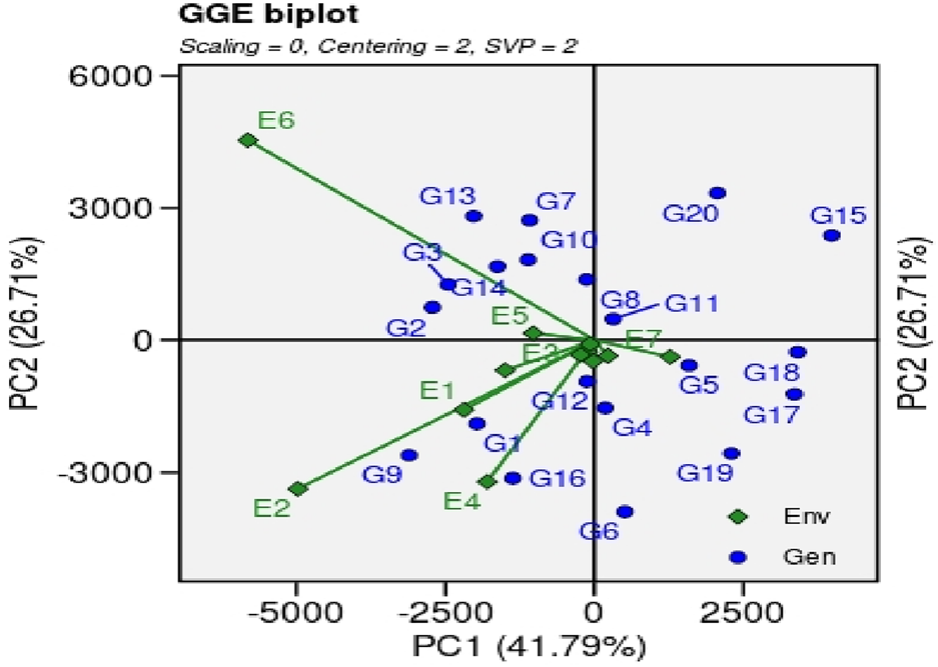
The GGE biplot design of genotype × environment interaction of 20 GSR lines and 2 check cultivars planted at 12 environments during the year 2021 for paddy yield. Note: METAN R package was used to generate figures.

**Figure 3:**
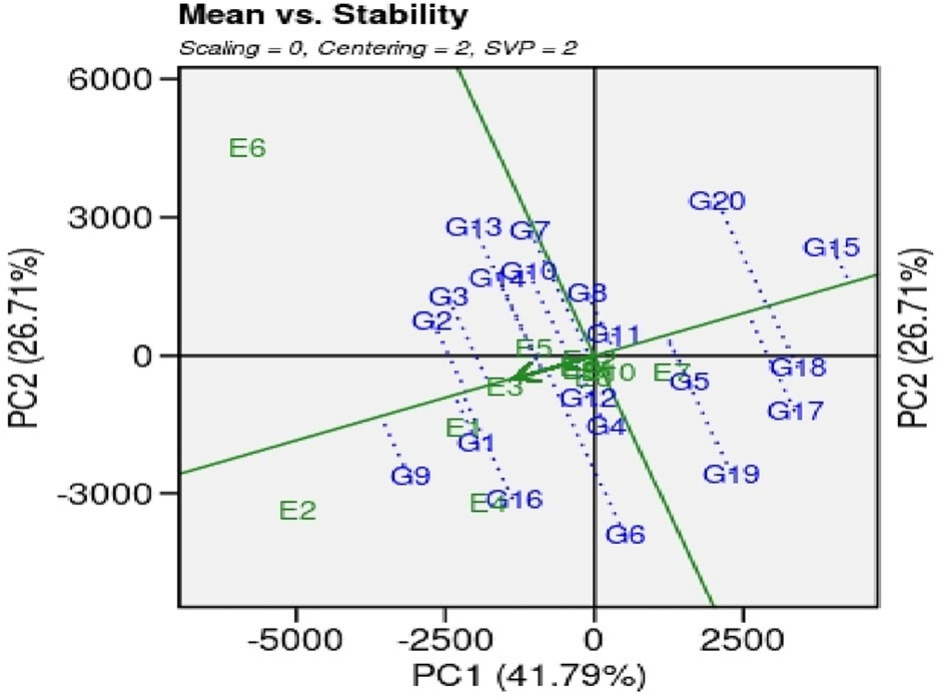
‘Mean versus stability’ GGE biplot of 20 GSR lines including checks planted at 12 environments during the year 2021 for paddy yield.

The stability pattern of GSR lines was assessed based on their mean performance across various environments. The mean *vs.* stability graph is generated by the intersection of vertical and horizontal ordinate lines (Figs. 2, 3). Every genotype has a single arrow that has direction towards a mean performance for trait under study. In the present study mean *vs.* stability analysis of GGE biplot revealed 68.5% variability for genotypes on the bases of paddy yield over multilocation. The genotypes, G2, G5, G11 and G18 revealed higher paddy yield in environments, E3, E5, E7, E8, E10, E11 and E12. The genotypes depicted less arrowhead lines to these environments and considered as a best performing genotype in almost all locations.

#### ‘Which-won-where’ GGE biplot

**Figure 4:**
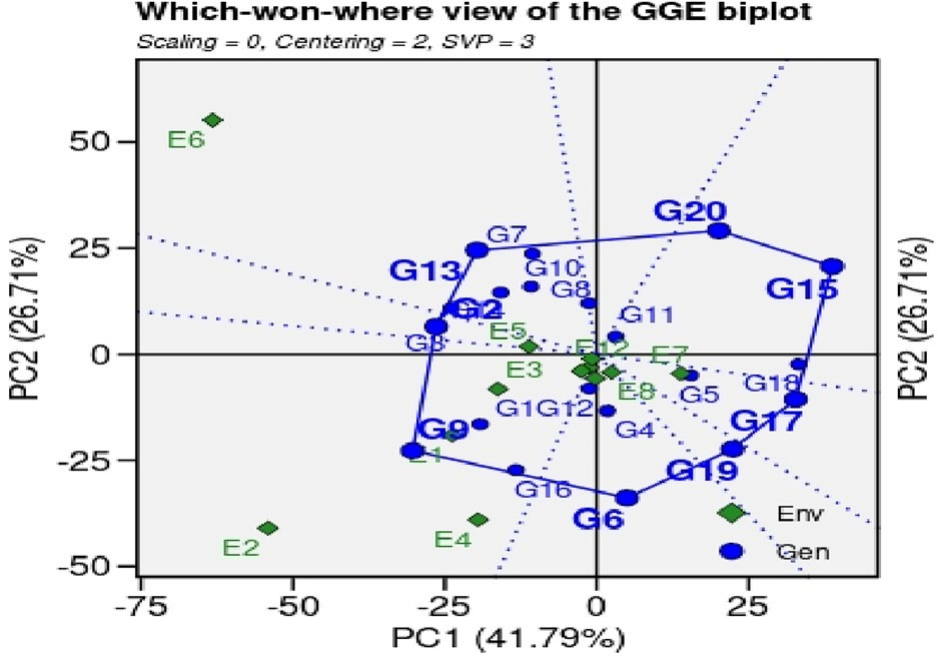
‘Which − won − where’ view of GGE biplot 20 GSR lines including checks under 12 environments during the year 2021 for paddy yield.

The GGE biplot is presented in polygon view in Fig. 4. It determined the best performing genotypes for paddy yield in a group of locations (Environments). The ‘which-won-where’ GGE biplot revealed 68.62% variability in first two PCs for paddy yield. The GGE biplot explained according to, as described by^50^. The genotypes present near the vertex of polygon with no environment nearby are considered less stable and genotypes present on the vertex of polygon where one or more environments are prevalent are considered best performing genotypes. The genotypes present inside the polygon are less responsive to tested environment and are considered the best performing genotypes over a broad range of environments. In Fig. 4 the polygon is divided into five sectors representing 12 environments. Sector-I contains E1, E4, E7 and E12 environments. Sector-II contains E3, E8, E9, E10, and E11 environments while Sector-III includes only one environment E5. Sector-IV is represented by E6 while Sector-V contains E2 and E4 environments. The genotype, G2, G13 and G7 were best performing genotypes in E6. Genotypes G9 and G16 were best-performing genotypes in tested environments, E2 and E4. The genotypes (G6, G14, G19, G17, G18, G15 and G20) are poorly performing genotypes because they are lying in an area with no representation of any environment. The genotypes (G1, G4, G5, G8, G10 and G14) that are less responsive to any environment and exhibited stable performance for yield in all the environments.

#### ‘Discriminativeness *vs.* representativeness’ pattern of stability

**Figure 5:**
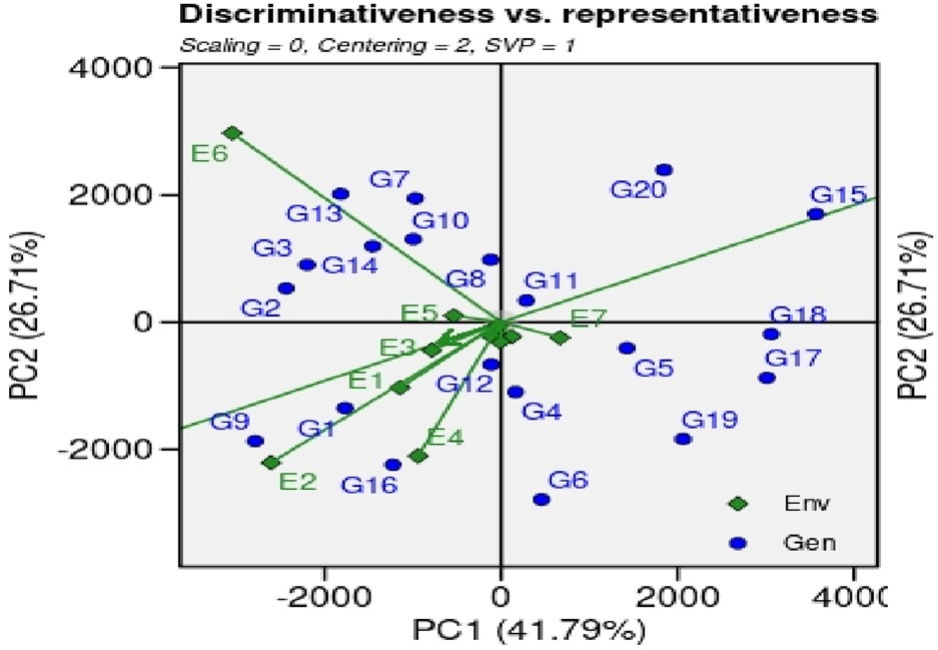
‘Discriminativeness vs. representativeness’ view of GGE biplot of 18 GSR lines and 2 check cultivars grown under 12 environments during the year 2021 for paddy yield.

The GGE biplot polygon is divided into four quadrants (Fig. 5). Genotypes, G11, G15 and G20 that fall in the upper right quadrant of the polygon have high discriminativeness and high representativeness and are considered the most desirable genotypes. Genotypes, G4, G5, G6, G18, G19 and G17 that fall in the lower right quadrant have high ‘discriminativeness and low representativeness’ and are suitable for specific environments or management practices. In this case G17 is the most suitable for these genotypes. Genotypes (G2, G3, G7, G8, G10, G13 and G14) in the upper left quadrant have low Discriminativeness and high representativeness, are best suited for average or low-yielding environments. In this case environments, E5 (D.I Khan, KPK) and E6 (Usta Muhammad, Baluchistan) were best suited environments for yield performance. Finally, genotypes located in the lower left quadrant have low discriminativeness and low representativeness and should be discarded.

#### IPCA and WAASB/GY ratio-based stability heat-map

**Figure 6:**
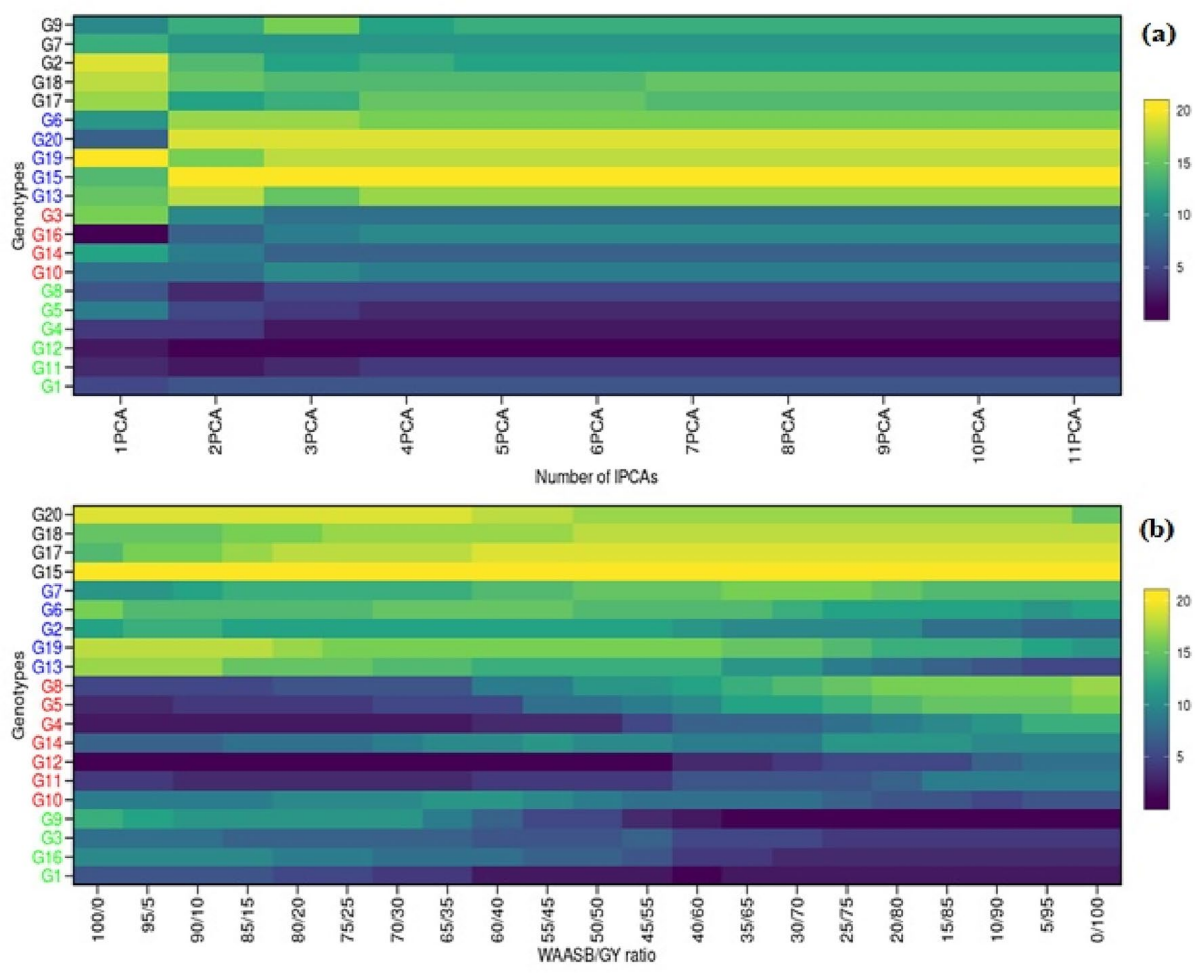
**a** Shows the genotype ranking depending on the number of principal component axis used for estimating the WAASB index. Four clusters of genotypes are shown by label colors (red) unproductive and unstable genotypes; (blue) productive, but unstable genotypes; (black) stable, but unproductive genotypes; and (green), productive and stable genotypes. **b** Shows the genotype ranking depending on the WAASB/GY ratio. The ranks obtained with a ratio of 100/0 consider exclusively the stability for the genotype ranking. On the other hand, a ratio of 0/100 considers exclusively the productivity for the genotype ranking.

Interaction Principal Components (IPCA) and weighted average of absolute Scores (WAASB) heat map (Fig. 6) is a graphical representation that uses color-coded cells to display data in a matrix format. In the context of stability analysis and the estimation of the WAASB (Weighted Average of Absolute Scores on Principal Component Axes) index, a heatmap can be used to show the genotype ranking based on the number of principal component (PC) axes utilized. *Axis Labels:* The horizontal axis of the heatmap represents the number of principal component axes used for estimating the WAASB index. It typically starts from a lower value and increases incrementally. The vertical axis represents the genotypes being analyzed. *Color Coding:* Each cell in the heatmap is color-coded to represent the genotype’s ranking based on the WAASB index. The colors may range from a gradient of a single color (e.g., lighter to darker shades of blue) or a spectrum of multiple colors (e.g., a rainbow palette). The color intensity or hue can indicate the relative ranking, with darker or more vibrant colors representing higher rankings and lighter or paler colors representing lower rankings. *Genotype Ranking:* The heatmap provides a visual representation of how different genotypes are ranked based on their WAASB index scores. The rows (vertical axis) correspond to individual genotypes, and the cells’ colors reflect their relative rankings across different numbers of PC axes. *Interpretation:* Observing the heatmap, one can identify patterns or trends in genotype rankings. For example, genotypes that consistently rank high across different PC axes will display cells with darker colors throughout the heatmap. Conversely, genotypes that consistently rank low will have cells with lighter colors. Inconsistencies or variations in rankings may be represented by cells with mixed colors or transitions between lighter and darker shades. Optimal Number of PC Axes: By examining the heatmap, researchers can assess the influence of the number of PC axes on genotype rankings. They can observe if there is a specific number of PC axes where genotypes consistently achieve higher rankings. Heat map revealed stable genotypes i.e., G8, G5, G4, G12, G11, G2, G3 and G1 based on IPCAs and WAASB.GY ratio.

#### Ranking environments

**Figure 7:**
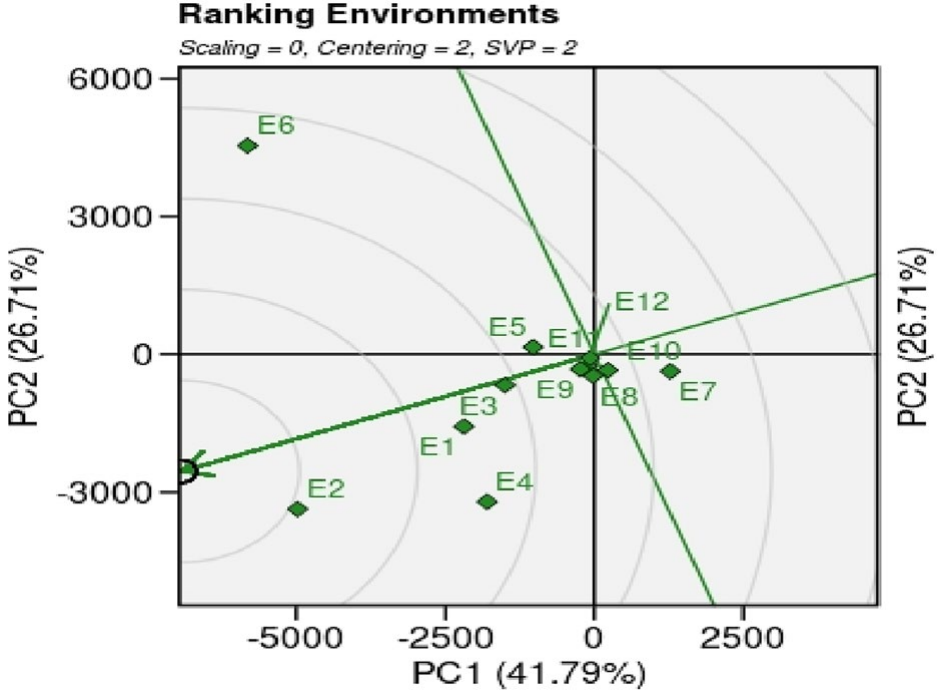
The GGE biplot ‘Ranking Environments’ profile to rank environments for the ideal location of 20 GSR lines including checks grown under 12 environments during the year 2021 for paddy yield.

The environment E2 (KSK, Lahore) is the best environment for paddy yield performance for all the genotypes tested (Fig. 7). The tested environment for paddy yield was revealed based on nearness to the concentric centers. In this biplot analysis, genotypes are considered as random samples of testing genotypes. The pattern of environment ranking for GGE biplot for paddy yield is E2 > E4 ≈ E1 > E1 > E3 > E5 ≈ E8 ≈ E ≈ E2 ≈ E4 ≈ E5 ≈ E9 ≈ E10 ≈ E11≈ E12 > E7 > E6.

#### Ranking of genotypes

**Figure 8:**
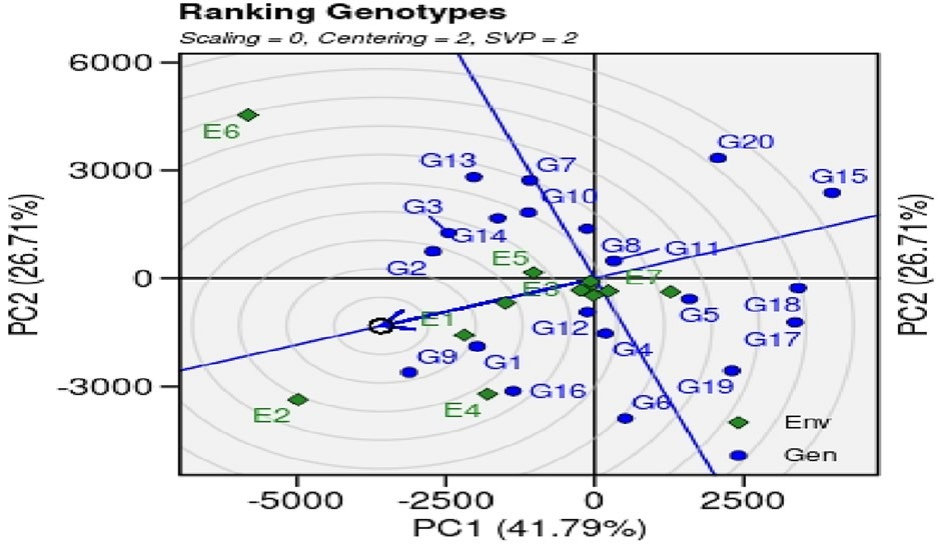
The GGE biplot ‘Ranking genotypes’ pattern to rank genotypes to find out the ideal genotype of 20 GSR lines including checks evaluated under 12 environments during the year 2021for grain yield.

The G + G × E biplot for genotype ranking revealed ideal genotype as compared to other genotypes under study (Fig. 8). The arrowhead indicates the ideo-types that performed well in all tested locations. The genotypes near to the concentric arrow are considered the best performing genotypes. In this biplot analysis locations are considered as random samples of testing environment. The pattern of genotypes ranking for GGE biplot for paddy yield is G9 ≈ G2 ≈ G14 ≈ G3 > G1 > G13 > G12 > G8 > G11 > G7 ≈ G10 ≈ G4 > G6 > G5 > G11 > G20 > G19 > G17 > G18 > G15.

### Discussion

Introduction of GSR is a success story in Pakistan after its yield performance we revealed at local climatic condition as compared to locally adopted check cultivars. Our findings for GSR paddy yield performance are in comparison with the results of previous studies by^51^. For variety development, GSR germplasm was tested over broad range of environmental conditions country wide in Pakistan. Multilocation yield trials are the major activity of breeding program to assess the genotypes or candidate line for stable performance and adaptability over the broad range of environmental conditions. Selection of genotype for multi-environment based on prediction values in comparison with observed values is crucial^44^. There are three main factors that can increase the accuracy of prediction of suitable genotypes in multi-environments (ME). The first one is the use of experimental design which includes the plot size, field management and area of experiment. The second one is the increased number of replications to minimize the experimental error. Third one is the statistical analysis that can predict accuracy in comparison with observed values for selection of suitable ideotype^52^.

From breeding point of view, the estimation of genotypic variance, phenotypic variance, environmental variance, interaction of G × E by analysis of variance and heritability estimates are very important in terms of gene expression controlling complex traits like grain yield over a broad range of environmental conditions. Based on information about variance contribution by genotypes and environment, an ideal genotype can be selected with less influence by environment. Only heritable variations transfer from generation to generation for a particular trait in a genotype that is essential for selection of suitable genotype^53^. A genotype with high broad sense heritability and genotypic variance is more stable as compared to genotypes with more influenced by environmental variance^54–56^. In the present study, analysis of variance revealed significant differences for all the traits studies (PH, PL, GPP, TGW and YLD) except NT, which was non-significant. Significant G x E interaction depicted that multi-environment (ME) played important role for the expression of genotypes for paddy yield and these similar finding was reported by^57^. Genotypic variance and broad sense heritability estimates were recorded in higher magnitude for all the traits except NT and similar findings were reported by^54,55^. However, careful selection of genotypes should be made while selection of genotypes for NT because this trait is governed by environmental variance.

From the agronomic point of view of AMMI biplot analysis, GEI biplot, ‘which-won-where’ biplots and WAASB facilitates selection of genotypes performed well in a particular environment^44^. In our study, we evaluated genotypes at multi-location, and data was subjected to both univariate and multivariate statistics. Univariate statistics such as ASI, ASV, *b*_*i*_, Wi^2^, σ^2^i and WASSB is used to identify the most stable genotypes based on certain parametric statistics. Based on ASV, ASI, *bi,* Wi^2^, σ^2^_i_ and WAAS, the genotypes G1, G4, G5, G8, G11 and G12 revealed the lowest values for all the stability statistics. These genotypes are considered the most stable genotypes based on these statistics and results obtained in this study are in accordance with the early findings^58^. Univariate stability statistics have some limitations as compared to multivariate statistics such as AMMI analysis and GGE biplot analysis^58^.

Multivariate stability statistics are often preferred due to the complex nature of rice production systems and the interdependencies among various factors affecting stability. Rice production and stability are influenced by multiple variables, such as climate, soil conditions, agronomic practices, and management strategies. Multivariate stability statistics can consider the joint effect of these factors, providing a more holistic view of stability compared to analyzing individual factors in isolation. Among multivariate methods, the AMMI analysis is mostly used for G x E interaction^54^. Non-parametric statistics such as the biplot mostly point out suitable genotypes in a particular location based on their performance. In our study, AMMI analysis of variance revealed significant GGE interaction and the first two Interaction Principal Component Axis (IPC1 and IPC2) were significant and depicted 39% and 67% variance for paddy yield, respectively (Table 6). This result indicates interaction of the environment had an important contribution to the performance of a genotype^53^.

Performance of genotypes for yield, agronomic and other botanical traits is influenced by multiple environmental factors such as photoperiod, temperature, humidity, darnel changes, soil types, rain, competitions with local biotic factors (weeds, diseases, insects, soil microbiomes interactions with plants, acidity/alkalinity of soil fertility status of soil, time of sowing etc. Keeping in view, various multivariate statistics i.e. ‘Mean *vs.* Stability’ analysis of GGE biplot, ‘Which-won-where’ GGE biplot, ‘Discriminativeness *vs.* representativeness’ pattern of stability, ‘ranking environments’, ‘ranking genotypes’ were used to identify higher yielder and stable genotypes in particular environments By using ‘Mean *vs.* Stability’ analysis of GGE biplot, the genotypes, G2, G5, G11 and G18 revealed higher paddy yield in environments, E3 (NIAB, Faisalabad), E5 (ARI. D.I Khan, KPK), E7 (RRS, Bahawalnagar, Punjab), E8 (RRI, Dohkri, Sind), E10 (Shaikhupura, Punjab), E11(Gujranwala, Punjab) and E12 (Shikarpur, Sind). According to the ‘Which-won-where’ GGE biplot, 12 environments were grouped into five sectors i.e., Sector-I (E1, E4, E7 and E12), Sector-II (E3, E8, E9, E10, and E11), Sector-III (E-5), Sector-IV (E6) and Sector-V (E2 and E4). These sectors are based on similar environmental conditions and the performance of genotypes in these environments. The genotypes, G2, G13 and G7 were the best performing genotypes in Sector-IV which contains only a single environment, E6 (Usta Muhammad, Baluchistan). These genotypes can be recommended in Usta Muhammad, Baluchistan area for cultivation after further testing and evaluations for yield, this environment has highly fertile soil and high temperature conditions. The genotypes G9 and G16 were best performing in Sector-V (SSRI, Pindi Bhattian, and KSK, Lahore, Punjab); these genotypes are best performing in alkaline soils because one of the environments i.e. E4 has highly saline soil conditions. The GGE biplot polygon is utilized to characterize the discriminativeness and representativeness of genotypes in multi-environment. The polygon characterized a graphical interface between genotypes and environments, where each point in the polygon corresponds to a genotype and represents its performance across different environments. The x-axis of the polygon represents discriminativeness which is a measure of the genotype's ability to differentiate among the tested environments. A genotype with high discriminativeness can be used to select the best performing genotypes across different environments. The y-axis of the polygon represents representativeness which is a measure of the genotype's stability across different environments. A genotype with high representativeness performs consistently in all environments, even if it does not necessarily have the highest yield. In case of the ‘Discriminativeness *vs.* representativeness’ pattern of stability, the genotypes, G2, G3, G7, G8, G10, G11, G13 and G14, G15, G17 and G20 that fall in the upper right quadrant of the polygon have high discriminativeness and high representativeness and are considered the most desirable genotypes. In this pattern stability study, we identify two environments, E5 (D.I Khan, KPK) and E6 (Usta Muhammad, Baluchistan) that were best-suited environments for yield performance under hot weather conditions. The genotypes that performed best in these two environments are likely to heat and drought resistant genotypes and should be recommended for cultivation under drought and high-temperature environments after farther testing for yield and agronomic traits.

According to the ranking environment, environment E2 (KSK, Lahore) is best environment for paddy yield performance for all the genotypes tested and ranking genotypes, the genotypes G1, G2, G8, G11, G12, G13 and G14 were identified as best performing genotypes in case of paddy yield. Heatmap (Fig. 7) also revealed stable genotypes i.e., G8, G5, G4, G12, G11, G2, G3 and G1 based on IPCAs and WAASB.GY ratio. The genotypes can be utilized for stable and high yielding variety development process and can be used in the future breeding program for high-yielding, salt, and drought tolerant variety development.

## Conclusion

Paddy yield in rice is a complex polygenic trait, governed by several factors. Environmental interaction of genotypes for the expression of genes related to yield is an important phenomenon. Testing of genotypes in a wide range of environmental conditions is a prerequisite before the commercial release of cultivar by plant breeders. To identify the most stable genotypes in a multi-environment, we evaluated 20 GSR lines including checks by using univariate and multivariate statistical approaches. Based on ASV, ASI, *bi,* Wi^2^, σ^2^_i_ and WAAS statistics, the genotypes G1, G4, G5, G8, G11 and G12 revealed lowest values for parametric statistics and observed more stable genotypes. To further validate the results, data was subjected to multivariate statistics and AMMI analysis. This revealed significant differences among genotypes and G x E interaction. GGE biplot depicted 67% variability among genotypes in the first two PCs. Based on GGE biplot analyses, ‘IPCA and WAASB/GY’ ratio-based stability Heat-map and ranking of genotypes, the genotypes namely G1, G2, G3, G5, G8, G10, G11 and G13 were observed better performing and stable. It is concluded from our study that these genotypes could be recommended for commercial cultivar development after further testing in national yield trials.

## Data Availability

The datasets generated and analyzed during the current study are available from the corresponding author on reasonable request.
